# Association of estimated glomerular filtration rate and proteinuria with all-cause mortality in community-based population in China: A Result from Kailuan Study

**DOI:** 10.1038/s41598-018-20554-3

**Published:** 2018-02-01

**Authors:** Jianwei Wu, Jiaokun Jia, Zhaoxia Li, Hua Pan, Anxin Wang, Xiuhua Guo, Shouling Wu, Xingquan Zhao

**Affiliations:** 1 Department of Neurology, Beijing Tiantan Hospital, Capital Medical University, Beijing, China; 2 China National Clinical Research Center for Neurological Diseases, Beijing, China; 3 Center of Stroke, Beijing Institute for Brain Disorders, Beijing, China; 4 Beijing Key Laboratory of Translational Medicine for Cerebrovascular Disease, Beijing, China; 5 Beijing Municipal Key Laboratory of Clinical Epidemiology, Beijing, China; 6 Department of Epidemiology and Health Statistics, School of Public Health, Capital Medical University, Beijing, China; 7 Department of Cardiology, Kailuan Hospital, Hebei United University, Tangshan, China

## Abstract

This study was based on 95391 participants (18–98 years old) from the Kailuan study, which assessed all-cause mortality in a community-based population in northern China according to estimated glomerular filtration rate (eGFR) by the Chronic Kidney Disease Epidemiology Collaboration (CKD-EPI) formula and proteinuria estimated from urine dipstick results. Data were analysed based on Cox proportional hazards models with adjustment for relevant confounders, and the results were expressed as hazard ratios (HRs) with 95% confidence intervals (CIs). During eight years of follow-up, a total of 6024 participants died. The two indicators, eGFR < 45 ml/min/1.73 m^2^ and the presence of proteinuria, were independently associated with all-cause mortality. Compared with eGFR ≥45 ml/min/1.73 m^2^ with negative proteinuria, HRs of all-cause mortality were 1.26 (95% CI 1.10–1.44) for eGFR < 45 ml/min/1.73 m^2^ without proteinuria, 1.95 (1.78–2.14) for eGFR ≥45 ml/min/1.73 m^2^ with proteinuria, and 2.63 (2.14–3.23) for eGFR < 45 ml/min/1.73 m^2^ with proteinuria. The all-cause mortality risk of eGFR and/or proteinuria was much higher in females than in males (P for interaction < 0.01). In conclusion, both severely decreased eGFR and proteinuria are independent predictors of all-cause mortality in the general northern Chinese population. A combination of severely decreased eGFR and proteinuria increases the risk of all-cause mortality, which is even over 5-fold higher in females.

## Introduction

The burden of chronic kidney disease (CKD), which is a worldwide public health problem with increasing prevalence, poor outcomes and high treatment costs, is substantial^1^. The disease affects 10–16% of the adult population in Asia, Europe, and the USA^2–4^, increases the risk of all-cause mortality and cardiovascular disease, and may further develop into kidney failure that is independent of other traditional risk factors^5,6^. According to WHO global health, it is estimated that 12.2 deaths per 100 000 people were attributable to CKD in 2012, and the death rate from CKD will continue to increase to reach 14 per 100 000 people by 2030^7,8^. Current international guidelines define CKD as 1) glomerular filtration rate (GFR) < 60 ml/min per 1.73 m² or 2) markers of kidney damage, or both, of at least a 3 month duration^9^. GFR is the best available indicator of overall kidney function^7^. Meanwhile, as a measure of kidney damage, proteinuria is associated with an increasing risk of early death, while early reductions in proteinuria are associated with a slower progression of kidney disease^10^.

Several studies have shown that decreasing estimated GFR (eGFR), increasing proteinuria and/or eGFR accompanied with proteinuria are associated with all-cause mortality in varying populations, including American, British, Canadian White, Chinese in Canada and South Asian in Canada^11–13^. Most studies, whose subjects were Chinese patients with specific diseases such as diabetes and stroke^14–16^, only showed the association between eGFR, proteinuria and all-cause mortality. Few studies have focused on the general Chinese population, except for one study, which suggested an independent association between the combination of eGFR and proteinuria and all-cause mortality in 462293 adults from a middle-aged working population of Taiwan Chinese^2^. However, eGFR, proteinuria and the combined relationship have not been examined carefully in a larger general population from northern China, where the living environment and dietary habits are diversely different from those in Taiwan.

Thus, in this study, whether eGFR, proteinuria and eGFR accompanied with proteinuria were associated with all-cause mortality in a large cohort of the northern Chinese population was examined.

## Results

### Baseline characteristics

A total of 95391 participants had available data for eGFR and urine dipstick protein assessments at baseline. Among these participants, the mean (±SD) age was 52.0 ± 12.6 years at entry, and 20.1% of the participants were women.

The baseline clinical and biochemical characteristics of participants by eGFR categories are shown in Table 1. The mean (±SD) age of participants with eGFR ≥90 ml/min/1.73 m^2^, 60–89 ml/min/1.73 m^2^, 45–59 ml/min/1.73 m^2^ and <45 ml/min/1.73 m^2^ were 47.13 ± 11.16, 53.23 ± 12.07, 58.98 ± 13.22 and 58.03 ± 14.61, respectively, the difference of which was significant. Additionally, compared with those with normal or near-normal eGFR, participants with lower levels of eGFR had higher body mass index, high-sensitivity C-reactive protein, low-density lipoprotein cholesterol, high-density lipoprotein cholesterol, triglycerides, and total cholesterol. At progressively lower baseline levels of eGFR, the prevalence was higher for current smoking and drinking, prior history of diabetes mellitus, hypertension, dyslipidaemia, myocardial infarction, stroke, hyperuricaemia and atrial fibrillation. Proteinuria was documented in a higher proportion of patients with lower eGFR levels (Table 1).

**Table 1:** Clinical characteristics of participants grouped by baseline estimated glomerular filtration rate (eGFR).

	Baseline eGFR (ml/min/1.73 m^2^)				P
	≥ 90 (n = 31129)	60–89 (n = 51109)	45–59 (n = 10702)	<45 (n = 2451)	
Gender (% female)	5415 (17.40)	10627 (20.79)	2544 (23.77)	587 (23.95)	<0.01
Age (years)	47.13 (11.16)	53.23 (12.07)	58.98 (13.22)	58.03 (14.61)	<0.01
Current smoker (%)	11399 (36.62)	17189 (33.63)	2446 (22.86)	521 (21.26)	<0.01
Current alcohol (%)	12505 (40.17)	18535 (36.27)	2485 (23.22)	541 (22.07)	<0.01
Body mass index (kg/m^2^)	24.66 (3.53)	25.18 (3.46)	25.56 (3.40)	25.59 (3.58)	<0.01
Systolic blood pressure (mmHg)	126.71 (19.29)	131.99 (20.99)	138.96 (22.13)	140.48 (22.81)	<0.01
Diastolic blood pressure (mmHg)	82.03 (11.40)	83.90 (11.79)	86.08 (11.98)	86.73 (12.16)	<0.01
Hypertension, n (%)	2467 (7.93)	6984 (13.66)	2019 (18.87)	447 (18.24)	<0.01
Diabetes mellitus, n (%)	637 (2.05)	1757 (3.44)	538 (5.03)	141 (5.75)	<0.01
Dyslipidaemia, n (%)	1198 (3.85)	3517 (6.88)	868 (8.11)	179 (7.30)	<0.01
Myocardial infarction, n (%)	271 (0.87)	698 (1.37)	238 (2.22)	58 (2.37)	<0.01
Stroke, n (%)	863 (2.77)	2073 (4.06)	551 (5.15)	141 (5.75)	<0.01
Atrial fibrillation, n (%)	68 (0.22)	230 (0.45)	113 (1.06)	23 (0.94)	<0.01
High-sensitivity C-reactive protein, mg/L	2.49 (5.83)	2.37 (7.03)	2.45 (6.09)	2.66 (6.72)	<0.01
Total cholesterol, mmol/L	2.49 (1.52)	2.52 (1.54)	2.56 (1.63)	2.78 (2.01)	<0.01
Triglycerides, mmol/L	2.33 (1.10)	2.31 (1.00)	2.41 (1.03)	3.01 (2.08)	<0.01
Low density lipoprotein cholesterol, mmol/ L	2.19 (0.95)	2.37 (0.91)	2.56 (0.85)	2.56 (0.81)	<0.01
High density lipoprotein cholesterol, mmol/ L	1.54 (0.42)	1.55 (0.40)	1.60 (0.40)	1.63 (0.43)	<0.01
Hyperuricaemia	1171 (3.78)	3350 (6.55)	849 (7.93)	349 (14.24)	<0.01
Proteinuria, n (%)	1063 (3.41)	1966 (3.85)	751 (7.02)	341 (13.91)	<0.01
Creatinine, μmol/L	71.60 (13.17)	94.68 (12.82)	118.81 (15.20)	190.94 (106.61)	<0.01
eGFR (ml/min/1.73 m^2^)	104.67 (28.88)	75.29 (8.35)	54.27 (4.04)	35.64 (9.38)	<0.01

Data are mean (SD) or number (%). P-values are for differences among groups. To convert creatinine from mg/dl to μmol/L, multiply by 88.4; to convert cholesterols to mg/dl, multiply by 38.67; to convert triglycerides to mg/dl, multiply by 88.6.

### Kidney function and all-cause mortality

**Figure 1:**
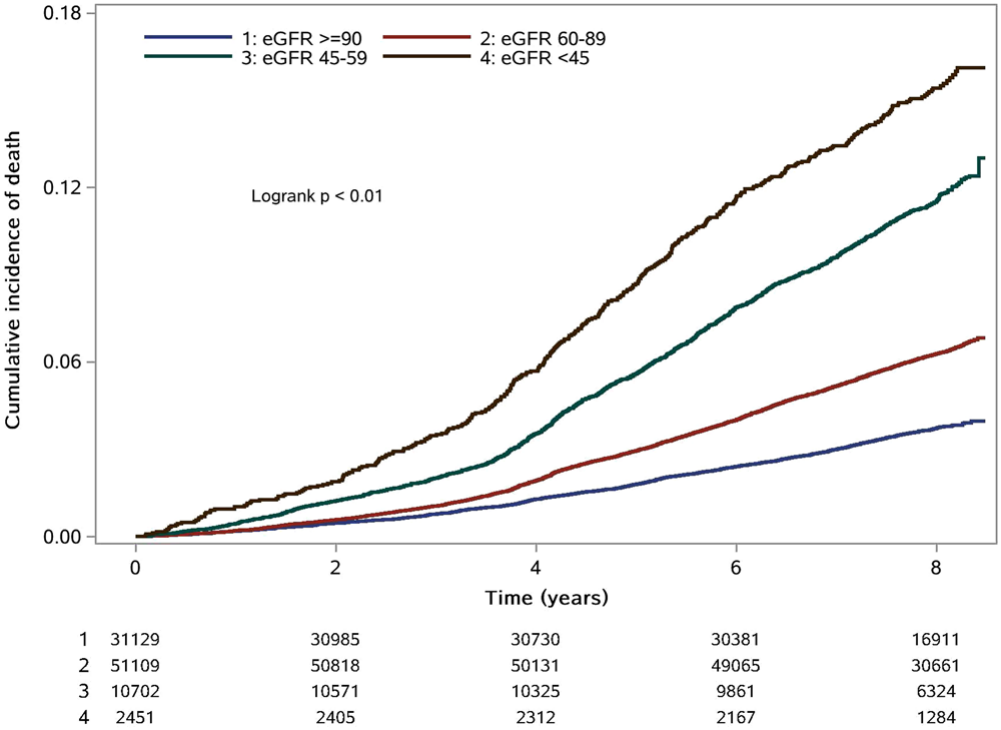
Kaplan–Meier survival curve for all-cause mortality stratified by eGFR levels.

**Figure 2:**
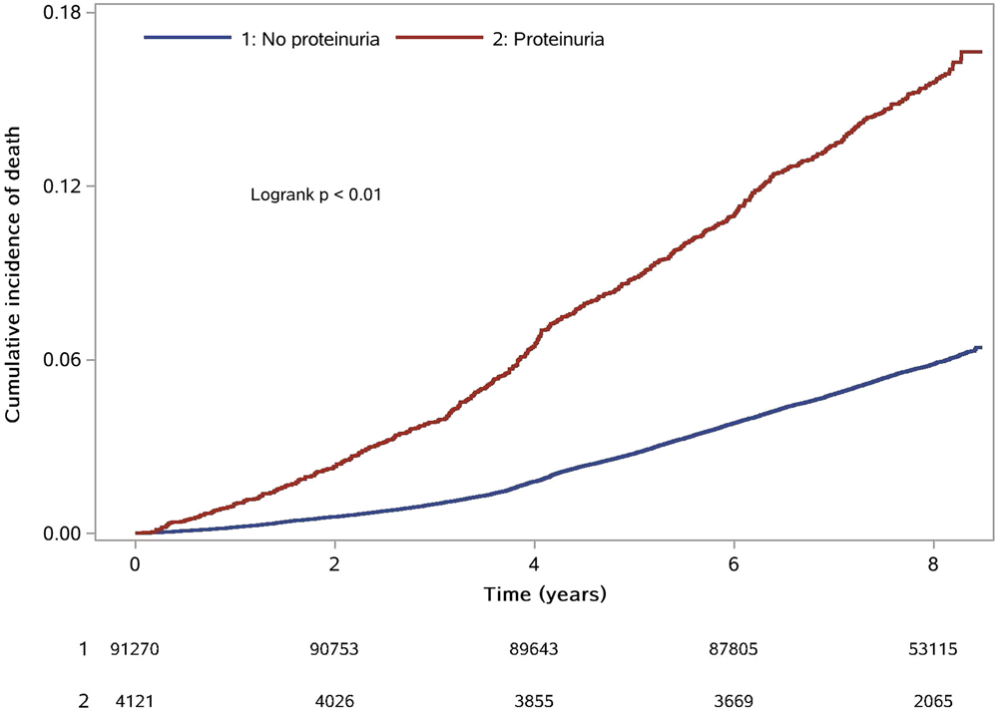
Kaplan–Meier survival curve for all-cause mortality stratified by proteinuria.

During the eight years of follow-up in this study, a total of 6024 participants died. The group of subjects with an eGFR of at least 90 ml/min/1.73 m^2^ was used as the reference group in the analysis of the association between the level of eGFR and all-cause mortality. In unadjusted regression analyses, the risk of all-cause mortality increased sharply as eGFR declined. Crude hazard ratios (HR) of all-cause mortality were 2.61 (95% CI 2.00–2.32) for the highest eGFR and 7.40 (95% CI 6.53–8.39) for the lowest eGFR (Table 2). After adjustment for age, gender, smoking status, drinking status, body mass index, high-sensitivity C-reactive protein, low-density lipoprotein cholesterol, high-density lipoprotein cholesterol, triglycerides, total cholesterol, and history of diabetes mellitus, hypertension, dyslipidaemia, atrial fibrillation, myocardial infarction, stroke and hyperuricaemia, the risk of all-cause mortality remained increased in the lowest eGFR category (HR 1.51, 95% CI 1.30–1.74, P < 0.01). Table 2 also shows that proteinuria significantly increased the risk of all-cause mortality in both univariate and multivariate regression analyses. The fully adjusted HR of all-cause mortality was 2.07 (95% CI 1.89–2.27, P < 0.01). Figures 1 and 2 show Kaplan–Meier survival curves stratified by eGFR levels and proteinuria, respectively.

**Table 2:** HR for the association of eGFR and proteinuria with the risk of all-cause mortality.

	eGFR(ml/min/1.73 m^2^)				P for trend	Proteinuria		P
	≥90	60–89	45–59	<45		No	Yes	
All-cause mortality (n = 6024)	n = 1155	n = 3230	n = 1259	n = 380		n = 5376	n = 648	
Crude model	Reference	2.16(2.00–2.32)	4.87(4.45–5.33)	7.40(6.53–8.39)	<0.01	Reference	2.89(2.67–3.14)	<0.01
Model 1	Reference	1.05(0.97–1.13)	1.20(1.09–1.33)	1.59(1.39–1.82)	<0.01	Reference	2.34(2.06–2.43)	<0.01
Model 2	Reference	1.01(0.93–1.09)	1.11(0.99–1.24)	1.51(1.30–1.74)	<0.01	Reference	2.07(1.89–2.27)	<0.01

Model 1 was adjusted for age and gender.

Model 2 was adjusted for age, gender, smoking status, drinking status, body mass index (BMI), hs-CRP, low-density lipoprotein cholesterol, high-density lipoprotein cholesterol, triglycerides, total cholesterol, and history of diabetic status, hypertension, dyslipidaemia, atrial fibrillation, myocardial infarction, stroke and hyperuricaemia by a Cox proportional hazards model.

**Figure 3:**
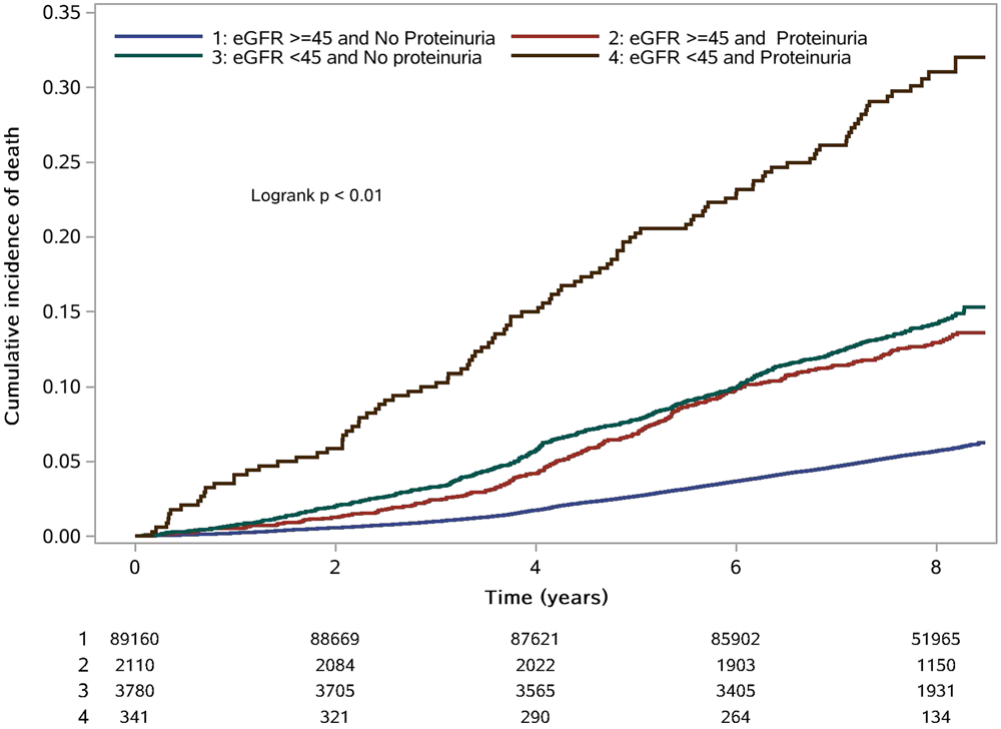
Kaplan–Meier survival curve for all-cause mortality stratified by eGFR and proteinuria (eGFR ≥45 + no proteinuria; eGFR ≥45 + proteinuria; eGFR < 45 + no proteinuria; eGFR < 45 + proteinuria).

Table 3 shows the combined relationship of proteinuria and the lowest eGFR with the risk of all-cause mortality. In this analysis, we found that compared with participants who had better eGFR (≥45 ml/min/1.73 m^2^) without proteinuria, the risks [HR(95%CI)] for participants with the lowest eGFR without proteinuria and eGFR ≥45 ml/min/1.73 m^2^ with proteinuria were 1.26 (1.10–1.44) and 1.95 (1.78–2.14), respectively. Moreover, the risk of eGFR < 45 ml/min/1.73 m^2^ with proteinuria was significantly higher than that of the other groups, such that the likelihood of dying was nearly 2.6-fold more than that of participants with eGFR ≥45 ml/min/1.73 m^2^ and negative urine protein (HR 2.63, 95% CI 2.14–3.23) (P < 0.01) (Fig. 3).

**Table 3:** Adjusted HR for all-cause mortality and cardiovascular events by eGFR and proteinuria.

Baseline eGFR (ml/min/1.73 m^2^)	≥45 HR (95%CI)	<45 HR (95%CI)	P for trend
All-cause mortality			<0.01
No proteinuria	Reference	1.26(1.10–1.44)	
Proteinuria	1.95(1.78–2.14)	2.63(2.14–3.23)	

Adjusted for age, gender, smoking status, drinking status, body mass index (BMI), hs-CRP, low-density lipoprotein cholesterol, high-density lipoprotein cholesterol, triglycerides, total cholesterol, and history of diabetic status, hypertension, dyslipidaemia, atrial fibrillation, myocardial infarction, stroke and hyperuricaemia by a Cox proportional hazards model.

Further analysis of the interaction of gender and hyperuricaemia on the association between eGFR/proteinuria and all-cause mortality showed that there was a significant difference between females and males (P for interaction < 0.01). The risk of all-cause mortality was increased in males with eGFR lower than 45 ml/min/1.73 m^2^ (HR 1.35, 95% CI 1.17–1.57); however, this risk was four times higher in females (HR 4.11, 95% CI 2.50–6.76). Similarly, the risk of getting proteinuria was higher for females than for males. However, these differences were not found in subjects with or without hyperuricaemia (P for interaction 0.27 or 0.66) (Table 4). Meanwhile, we also analysed the interaction of the combined relationship between proteinuria and the lowest eGFR with the risk of death (Table 5), which once again suggested that the difference was significant between genders (P for interaction < 0.01), and in female, who had the lowest eGFR combined with proteinuria, the risk of all-cause mortality was over 5-fold more than that in individuals who had eGFR ≥45 ml/min/1.73 m^2^ and negative proteinuria (HR 5.16, 95% CI 2.96–9.02). Whether or not participants have hyperuricaemia might affect the association between 1) the combination of the lowest eGFR and proteinuria and 2) death for any reason (P for interaction 0.32).

**Table 4:** Multivariate-adjusted HR and 95% CI for all-cause mortality according to eGFR or proteinuria, stratified by gender and hyperuricaemia.

	eGFR (ml/min/1.73 m^2^)				P for interaction	Proteinuria		P for interaction
	≥90	60–89	45–59	<45		No	Yes	
Gender					<0.01			<0.01
Female	Reference	1.65(1.16–2.34)	1.92(1.25–2.96)	4.11(2.50–6.76)		Reference	3.22(2.39–4.34)	
Male	Reference	1.01(0.94–1.10)	1.11(0.99–1.23)	1.35(1.17–1.57)		Reference	1.93(1.76–2.12)	
Hyperuricaemia					0.27			0.66
No	Reference	0.94(0.69–1.28)	1.04(0.73–1.49)	1.32(0.88–1.97)		Reference	2.00(1.82–2.20)	
Yes	Reference	1.04(0.96–1.13)	1.14(1.02–1.27)	1.53(1.32–1.79)		Reference	2.06(1.64–2.60)	

Adjusted for age, gender, smoking status, drinking status, body mass index (BMI), hs-CRP, low-density lipoprotein cholesterol, high-density lipoprotein cholesterol, triglycerides, total cholesterol, and history of diabetic status, hypertension, dyslipidaemia, atrial fibrillation, myocardial infarction, stroke and hyperuricaemia by a Cox proportional hazards model.

**Table 5:** Multivariate-adjusted HR and 95% CI for all-cause mortality according to eGFR and proteinuria, stratified by gender and hyperuricaemia.

	eGFR (ml/min/1.73 m^2^)		P for interaction
	≥45	<45	
Gender			<0.01
Female			
No proteinuria	Reference	2.12(1.45–3.12)	
Proteinuria	3.11(2.22–4.37)	5.16(2.96–9.02)	
Male			
No proteinuria	Reference	1.18(1.02–1.36)	
Proteinuria	1.89(1.71–2.09)	2.40(1.93–3.00)	
Hyperuricaemia			0.32
No			
No proteinuria	Reference	1.23(0.89–1.70)	
Proteinuria	2.06(1.59–2.67)	2.29(1.49–3.51)	
Yes			
No proteinuria	Reference	1.29(1.12–1.50)	
Proteinuria	1.94(1.75–2.14)	2.85(2.25–3.61)	

Adjusted for age, gender, smoking status, drinking status, body mass index (BMI), hs-CRP, low-density lipoprotein cholesterol, high-density lipoprotein cholesterol, triglycerides, total cholesterol, and history of diabetic status, hypertension, dyslipidaemia, atrial fibrillation, myocardial infarction, stroke and hyperuricaemia by a Cox proportional hazards model.

## Discussion

In this large, prospective study of the northern Chinese general population, the lowest category of eGFR was independently related to all-cause mortality and proteinuria. Additionally, a severe decrease in eGFR (<45 ml/min/1.73 m^2^) combined with proteinuria was associated with an increased risk of mortality, and the risk was nearly 2.6-fold higher than that for participants with better eGFR (≥45 ml/min/1.73 m^2^) without proteinuria, independent of traditional cardiovascular risk factors such as diabetes, hypertension, dyslipidaemia, atrial fibrillation, myocardial infarction, stroke and hyperuricaemia. Additionally, the lowest eGFR (<45 ml/min/1.73 m^2^) and/or proteinuria in females should be paid more attention, as there is a significantly higher risk of all-cause mortality in females than in males. To the best of our knowledge, this study is one of the first studies to assess the association between eGFR, proteinuria, and especially, the combined effect of these two indicators and the risk of all-cause mortality in a large northern Chinese general population.

These findings confirmed and extended prior studies. Prior population-based studies have shown that decreased eGFR or the presence of overt proteinuria were independently related to increased all-cause mortality^17–19^. Results from a collaborative meta-analysis conducted by the Chronic Kidney Disease Prognosis Consortium suggested that eGFR lower than 60 ml/min/1.73 m² is an independent predictor of mortality risk in the general population^20^. Meanwhile, the relationship was confirmed in 17026 adults, mostly aged 50 years or more, from Taiwan, China. The study divided participants into 3 groups by baseline eGFR categories, namely, ≥90, 60–89 and <60 ml/min/1.73 m², which also drew the conclusion that the population with eGFR lower than 60 ml/min/1.73 m² had a higher risk for all-cause mortality^21^. However, the conclusions from the above studies were a lack of distinction between eGFR <45 ml/min/1.73 m² and 45 to 60 ml/min/1.73 m², which might overstate the cut-off point of eGFR to predict all-cause mortality. A prospective cohort study involving multi-ethnic individuals, such as Chinese, Malay and Indian adults, from the Singapore Prospective Study Program and the Singapore Malay Eye Study concluded that eGFR lower than 45 ml/min/1.73 m² was independently associated with all-cause mortality, for which the risk was just slightly higher in participants with eGFR from 45 to 59.9 ml/min/1.73 m² than in participants with eGFR ≥60 ml/min/1.73 m², and there was no statistical significance. Similar to our results, after adjusting for potential covariates, there was no association between eGFR (between 45 ml/min/1.73 m² and 60 ml/min/1.73 m²) and all-cause mortality in the northern Chinese population. Additionally, compared with normal eGFR, only the risk for eGFR lower than 45 ml/min/1.73 m² is increasing, which suggests that 45 ml/min/1.73 m² but not 60 ml/min/1.73 m² might be the cut-off point for eGFR as the independent predictor of all-cause mortality for a population in northern China. Moreover, previous studies illustrated that all-cause mortality significantly increases along with the presence of proteinuria in the general population, independent of confounders^20,22–24^, which was also confirmed in the population from northern China. Several pathophysiologic mechanisms have been put forward as the reasons for poor clinical outcomes in patients with proteinuria, including diffused endothelial dysfunction, a reduction in the bioavailability of nitric oxide, an activated renin-angiotensin system, impaired endogenous thrombolytic activity, increased oxidative stress, and enhanced thrombogenicity^25,26^.

Meanwhile, several studies suggested that declining eGFR was associated with mortality in elderly females from America and Sweden^17,27^. Although the risk of death was unlikely to be different between genders in a Japanese population, the results only indicated an increased risk of mortality in females with eGFR < 45 ml/min/1.73 m^2^ ^28^. Our study also showed that when eGFR was lower than 45 ml/min/1.73 m², or when proteinuria was positive, the risk of all-cause mortality was much higher in northern Chinese females than in males. However, there was no difference in mortality risk between participants with hyperuricaemia and normal uric acid in northern China. In conclusion, this study, similar to previous studies, confirmed the association between eGFR, proteinuria and all-cause mortality. Additionally, this study provided clear evidence of the association in the northern Chinese general population.

Moreover, a cohort study from Canada showed that the risk of death from any cause in CKD patients was lower in Chinese individuals than in South Asian and White individuals living in Canada^12^. The combined relationship of eGFR and proteinuria with all-cause mortality was also found in a general population from Korea, and the risk of mortality in patients with decreased eGFR combined with proteinuria was nearly 4-fold higher than that of normal participants^29^. The African American Study of Kidney Disease Trial, which enrolled 1094 patients with hypertensive kidney disease and a median follow-up of 48.6 months, showed that the risk of all-cause mortality in participants with low GFR and high proteinuria was significantly higher than the risk in those with low GFR and low proteinuria, or with high GFR and high proteinuria, or with high GFR and low proteinuria without doubt^30^. It is also useful that eGFR and proteinuria were measured to predict the mortality rate in a Japanese population and future hypertension in both young and middle-aged males^31,32^. A Taiwanese study showed that the risk for all-cause mortality with proteinuria and decreased eGFR was approximately 2- to 10-fold higher that in than participants who had higher eGFR combined with negative proteinuria^2^. Similarly, from our study, the analysis of the combined relationship of eGFR and proteinuria suggested that, compared with the participants with better eGFR and negative urine protein, the risk of all-cause mortality was increased nearly 2.6-fold in those with severely decreased eGFR and proteinuria in the northern Chinese general population, and it was significantly higher than the risk in those solely with decreased eGFR or proteinuria. This tendency could be found in both genders, especially in females. Females with the lowest eGFR and proteinuria were over 5-fold more likely to die from any cause, which was significantly higher than the risk for males. Hence, decreased eGFR combined with the presence of proteinuria was a remarkable prediction of all-cause mortality, especially in females, and this study provided the evidence for a northern Chinese population.

This study has several strengths, including the prospective design, large sample size, long-term follow-up of the northern Chinese population, enrolment of both genders, standardized evaluation of directly measured body size, broad assessment of potential confounders, and confirmation of all-cause death through a review of medical records. Compared with other studies in Asian populations, this is the first study to assess both the independent and combined effects of reduced eGFR and the presence of proteinuria on all-cause mortality in a large northern Chinese general population. However, our study has some limitations. First, the participants in the Kailuan study do not constitute a nationally representative sample, and our findings may not be generalized directly to other Chinese populations with different educational and cultural backgrounds. Second, we used only a single urine collection for the assessment of proteinuria. However, proteinuria measured in a spot urine sample varies from day to day.

In conclusion, our findings suggest that both severely decreased eGFR and proteinuria can individually predict all-cause mortality in the general population of northern China, especially in females, independent of numerous confounding factors. The risk of all-cause mortality is 2.6-fold higher in participants with severely decreased eGFR combined with proteinuria than in those with better eGFR combined with negative urine protein, and the risk is over 5-fold higher in females.

## Methods

### Study design and population

The data for this study were abstracted from the Kailuan study. The study population and methodology have been previously reported in detail^33,34^. The Kailuan study was a prospective, population-based cohort study involving 101510 men and women aged 18–98 years in the Kailuan community in Tangshan City. In summary, all participants provided written informed consent and underwent questionnaire assessments, clinical examinations, and laboratory assessments. The protocol was approved by the Ethics Committee of both Kailuan General Hospital and Beijing Tiantan Hospital, in compliance with the Declaration of Helsinki.

In this study, 6119 participants were excluded from the analysis due to a lack of eGFR/proteinuria and other relevant data at baseline. As a result, 95391 participants, including 76218 men and 19173 women, were included in this analysis.

### Measurement of kidney function, proteinuria and estimated glomerular filtration rate (eGFR)

#### Proteinuria

Proteinuria was diagnosed by urine dipstick analysis using an automated dipstick urinalysis (H12-MA, DIRUI N-600), which was performed on a fresh urine sample by 3 physicians and read visually for 1 minute right after the dipstick test. The urine test strip results were based on a colour scale that quantified proteinuria as none, trace, 1+, 2+, or 3+ and we defined proteinuria as 1+ or greater protein. Women having menstrual periods were studied at a time away from the time of their periods. Pregnant women and subjects with active urinary tract infections were excluded.

### Estimated glomerular filtration rate (eGFR)

Blood samples were collected from an antecubital vein of participants in the morning after an overnight fast (>8 hours) and then transferred into vacuum tubes containing EDTA (Ethylene Diamine Tetraacetic Acid). All blood samples were processed and analysed using an auto-analyser (Hitachi 747; Hitachi, Tokyo, Japan) in the central laboratory of Kailuan General Hospital. The serum creatinine levels were measured by means of an enzymatic method on a Hitachi 7600 P auto-analyser (Hitachi, Tokyo, Japan). We calculated eGFR by using a modified 4-variable Chronic Kidney Disease Epidemiology Collaboration (CKD-EPI) formula with an adjusted coefficient of 1.1 for the Chinese population to estimate eGFR^35^: eGFR_CKD-EPI_ = 141 × min (SCr/κ,1)^α^ × max (SCr/κ,1)^−1.209^ × 0.993^Age^ × 1.018 (if female) × 1.1, where SCr was serum creatinine, κ was 0.7 for females and 0.9 for males, α was −0.329 for females and −0.411 for males, min was the minimum of SCr/κ or 1, and max indicated the maximum of SCr/κ or 1. The eGFR values were divided into four categories, <45, 45 to 59, 60 to 89, and ≥90 ml/min/1.73 m^2^, which were based on the National Kidney Foundation’s Kidney Disease Outcomes Quality Initiative (NKFK/DOQI).

### Assessment of potential covariates

Information on demographic variables (e.g., age, gender, smoking, and drinking status) was collected via questionnaires as described previously^34^. Body mass index is the ratio of body weight (kilograms) to height squared (square metres). Hypertension was defined as systolic blood pressure ≥140 mmHg or diastolic blood pressure ≥90 mmHg, any use of antihypertensive drugs, or any self-reported history of hypertension. Diabetes was defined as a fasting glucose level ≥7.0 mmol/L, non-fasting glucose concentration ≥11.1 mmol/L, any use of glucose-lowering drugs, or any self-reported history of diabetes. Dyslipidaemia was defined as serum triglyceride ≥150 mg/dl, low-density lipoprotein cholesterol ≥140 mg/dl, high-density lipoprotein cholesterol ≤ 40 mg/dl, any use of lipid-lowering drugs, or any self-reported history of dyslipidaemia. Hyperuricaemia was defined as any self-reported history of hyperuricaemia, or serum uric acid levels ≥420 μmol/L (7 mg/dl).

### Follow-up and outcome assessment

The participants were followed-up with face-to-face interviews every two years at routine medical examinations until December 31, 2014, or until death. The follow-ups were performed by hospital physicians, research physicians, and research nurses. The outcome information for participants without face-to-face follow-ups was obtained by referring to death certificates from provincial vital statistics offices, discharge summaries from the 11 hospitals, and medical records from medical insurance^33,34^.

All-cause mortality was defined as death from any cause, which was confirmed by either a death certificate from the local citizen registry or the record of the treating hospital. When no official documentation was available, case fatality was decided if death was reported on two consecutive follow-up periods by different proxies.

### Statistical analysis

Means, SD, and percentages were used to describe the baseline characteristics. We used multivariable Cox proportional hazards models to examine the association between proteinuria, eGFR and all-cause mortality during follow-ups. Multivariable models were adjusted based on age, gender, smoking status, drinking status, body mass index, C-reactive protein, low density lipoprotein cholesterol, high density lipoprotein cholesterol, triglycerides, total cholesterol, and history of diabetes mellitus, hypertension, dyslipidaemia, atrial fibrillation, myocardial infarction, stroke and hyperuricaemia. We included eGFR in the model as a categorical variable (≥90 [reference], 60 to 89, 45–59, and <45 ml/min/1.73 m^2^) and documented proteinuria as a dichotomous variable. We also examined the relationship of combined eGFR with proteinuria and the risk of all-cause mortality in a separate multivariable model. Additionally, gender and hyperuricaemia were evaluated to assess if there was any significant interaction between these variables and the relationship between eGFR or/combined with proteinuria and all-cause mortality. The estimated hazard ratios (HRs) and 95% confidence intervals (95% CIs) of variables were derived from the coefficient and standard error as determined by the Cox proportional hazards model. Values of P < 0.05 were accepted as indicative of statistical significance. Data were analysed using SAS version 9.4 (SAS Institute, Cary, North Carolina).
